# Update on pleural diseases - 2007

**DOI:** 10.4103/1817-1737.33704

**Published:** 2007

**Authors:** Ayman Bishay, Suhail Raoof, Adebayo Esan, Arthur Sung, Siraj Wali, Leonard Y. Lee, Liziamma George, Anthony Saleh, Michael Baumann

**Affiliations:** * *Division of Pulmonary and Critical Care Medicine, New York Methodist Hospital, Brooklyn NY, USA*; ** *Department of Internal Medicine, New York Methodist Hospital, Brooklyn NY, USA*; *** *Respiratory Section, King Khaled National Guard Hospital, Jeddah, Saudi Arabia*; **** *Department of Cardiothoracic Surgery, New York Methodist Hospital, Brooklyn NY, USA*; ***** *University of Mississippi Medical Center and University of Mississippi School of Medicine, USA*

**Keywords:** Pleural, empyema, effusions, mesothelioma, ultrasound, pleurodesis, pneumothorax, thoracoscopy

## Abstract

**BACKGROUND:**

New information is available on pleural diseases. The authors selected articles to make recommendations on diagnostic and treatment aspects of pleural diseases.

**MATERIALS AND METHODS:**

Eleven articles published in the English language between 2004 and 2007 were chosen. The basis of selection of the articles was the impact on daily practice, change in prior thinking of a disease process or specific treatment modality, as well as proper design and execution of the study. 5-amino-laevulinic acid with fluorescent light combined with white light may allow further diagnostic yield in undiagnosed pleural disease. FDG-PET may allow prognostication of patients with pleural tumors. Utilizing ultrasound by trained Emergency Department physicians is a rapid and effective technique to evaluate non-traumatic pleural effusions in symptomatic patients. Serum osteopontin levels may distinguish patients exposed to asbestos with benign disease from those with pleural mesothelioma. Administration of streptokinase in patients with empyema does not need for surgical drainage, length of hospital stay, or mortality as compared to conventional treatment with chest tube drainage and intravenous antibiotics. Silver nitrate may be an alternative agent to talc for producing pleurodesis. Routine use of graded talc (50% particles greater than 25 microns) is recommended to reduce the morbidity associated with talc pleurodesis. Study design does not permit us to conclude that aspiration of spontaneous pneumothorax is as effective as chest tube drainage. Pleural catheter may prove to be an important palliative modality in treating debilitated patients or patients with trapped lung who show symptomatic improvement with drainage; however, at the present time, these catheters cannot be considered a first line treatment option for patients with malignant pleural effusion. One of the studies reviewed showed no significant difference in tract metastasis in patients with malignant mesothelioma undergoing an invasive pleural procedure with or without irradiation to the procedure site. However, the design of the trial does not allow us to make this conclusion at the present time.

Diseases of the pleura may stem from primary lung diseases or result from consequences of systemic disorders. A plethora of infectious, inflammatory and neoplastic conditions may result in the formation of pleural effusions. Neoplastic disorders can cause effusions due to obstruction of the lymphatic channels or directly due to tumor metastasis to the pleural surfaces. These disorders may result in significant physiological impairment, leading to symptoms such as shortness of breath or pain and discomfort. It is therefore important to expediently establish the etiology of effusions, in order to address further therapeutic, prognostic and palliative issues. In the past, the diagnostic options available to the clinician were limited. Thoracentesis, closed pleural biopsy and thoracotomy via open surgical approach were the usual choices in the diagnostic armamentarium of the treating physician. Over the last few years, pulmonologists and intensivists are acquiring skills to utilize ultrasound to diagnose small or loculated pleural effusions, as well as to guide in the performance of some pleural procedures. Pleural manometry during thoracentesis allows the clinician to measure lung elastance, differentiating between normal lung and trapped lung physiology. This strategy improves patient safety when a large-volume thoracentesis is being performed, minimizing the potential for re-expansion pulmonary edema. Medical thoracoscopy [Figure 1] provides a direct view of the pleura and allows biopsy of localized and previously elusive disease [Figure 2]. In the same session, both diagnostic material may be obtained and pleurodesis performed.

**Figure 1:**
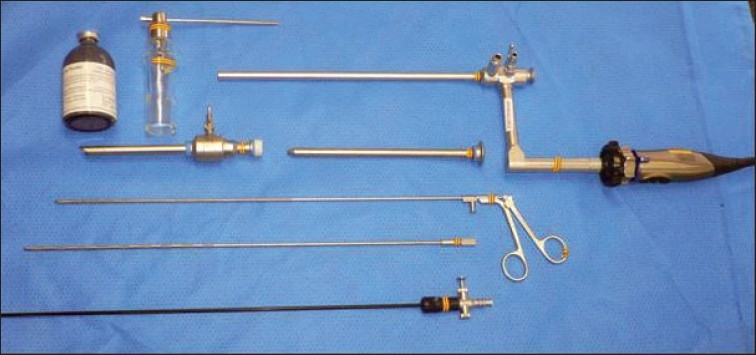
A medical thoracoscopy set (rigid) consists of a telescope that can accommodate a biopsy forceps, a probe and suction catheter (bottom). A blunt-tip trocar is used to introduce the cannula into the pleural space. A talc bottle with a powder blower is illustrated in the upper left aspect of the figure

**Figure 2:**
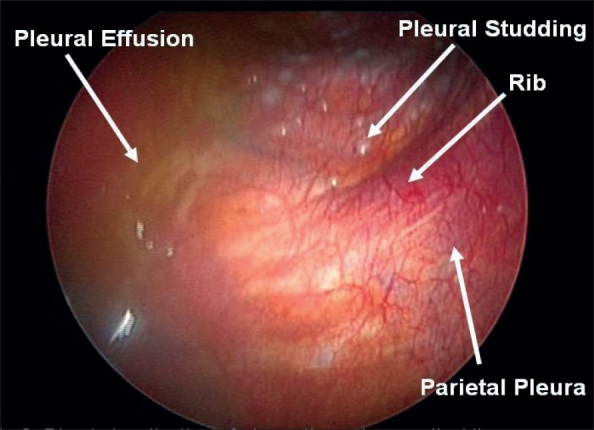
Medical thoracoscopy demonstrating metastatic breast cancer. Components of pleural effusion, as well as pleural studding of tumors, are seen on the surface of parietal pleura. Rib structures provide landmarks and orientations during the procedure

For the purpose of this review, we have summarized some of the more important clinical studies published in the English language between 2004 and 2007. The studies chosen cover different aspects of diagnosis and treatment of pleural diseases, with important clinical implications. The basis of selection of articles is as follows:

- **This article is likely to change my daily practice**
- **This article will change prior understanding or evidence of a disease process or treatment**
- **The study is well designed and executed**

A search was conducted utilizing Ovid and Pub Med. Eleven articles were selected and are discussed below briefly. The review consists of two categories of studies: those groups of studies discussing diagnostic modalities and those that focus on the treatment options of different pleural diseases.

**I. Diagnosis**

- Fluorescence detection of pleural malignancies using 5-aminolaevulinic acid
- Positron emission tomography predicts survival in malignant pleural mesothelioma
- Emergency ultrasound evaluation of symptomatic nontraumatic pleural effusions
- Asbestos exposure, pleural mesothelioma and serum osteopontin level

**II. Treatment**

- Fibrinolytics
  1. U.K. controlled trial of intrapleural streptokinase for pleural infection-multicenter intrapleural sepsis trial (MIST 1)
  2. Intrapleural fibrinolytic agents for empyema and complicated parapneumonic effusions: A meta-analysis
- Agents for pleurodesis
  1. Prospective randomized trial of silver nitrate *vs.* Talc Slurry in pleurodesis for symptomatic malignant pleural effusions
  2. Randomized trials describing lung inflammation after pleurodesis with Talc of varying particle size
  3. Simple aspiration *vs.* Chest tube insertion in the management of primary spontaneous pneumothorax: A systematic review
  4. Single-center experience with 250 Tunneled pleural catheter insertions for malignant pleural effusions
  5. Randomized trial of single-dose radiotherapy to prevent procedure tract metastasis by malignant mesothelioma

## I. DIAGNOSIS

### Fluorescence detection of pleural malignancies using 5-aminolaevulinic acid[1]

#### Background

Video-assisted thoracoscopy (VATS), with repeat pleural fluid examinations, has become a standard procedure in patients who have undiagnosed pleural disease. In patients with malignancies, VATS has aided in proper staging and has replaced the more invasive thoracotomy. In order to enhance intraoperative detection of subclinical tumor involvement of pleural surfaces, fluorescence methods have been studied for over two decades with the goal of augmenting the optical contrast of abnormal (malignant) tissue over normal surrounding tissue. It is suggested that fluorescence diagnosis (FD) using 5-aminolaevulininc acid (5-ALA) can improve the diagnostic yield during VATS.[2–4]

#### Methods

All eligible patients were enrolled for this study from January 2003 to January 2005. The study was conducted prospectively, and each of the selected patients with nondiagnostic pleural effusion was given oral doses of 5-ALA (1,500-2,500 mg based on patient weight) prior to undergoing VATS. Patients underwent inspection by both white light and fluorescence thoracoscopy, during which biopsy specimens were obtained from normal and abnormal sites for histological examination. Patients were discharged from the hospital 1-2 days after the procedure.

#### Results

**Figure 3:**
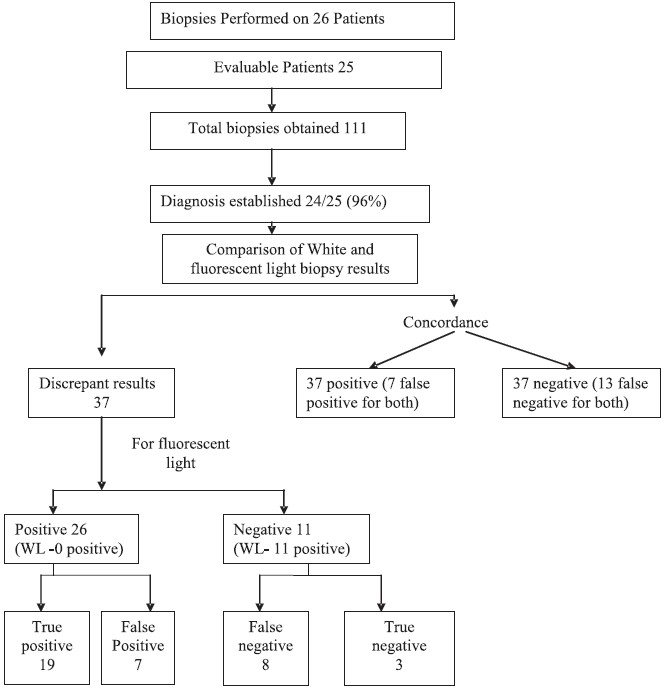
Results of biopsy specimens obtained utilizing white and fluorescent light. Out of the 111 biopsy specimens obtained, there was concordance amongst 74 specimens. The remaining 37 specimens showed discrepant results when obtained under white and fluorescent lights. *WL: White Light*

A total of 26 patients (17 men and 9 women) were enrolled, with 1 patient being excluded from analysis because thoracic cavity inspection could not be performed. The procedure lasted 30-90 min (median, 45 min), and only 2 patients had procedure-related complications. A diagnosis was obtained in 24 (96%) of the 25 patients. The spectrum of diseases included malignant mesothelioma (15 patients), metastasis from other tumors (5 patients), plaques with or without inflammatory changes (3 patients) and empyema (1 patient). A total of 111 biopsy specimens were obtained from different pleural sites. In 37 biopsies, results were positive in both FD and white light imaging, with 7 being false positives as the biopsies were from normal pleura or plaque. In another 37 biopsies, results were negative in both FD and white light imaging, with 13 being false negatives as the biopsies were found to be from tumor or proliferating nests of cells. There was a discrepancy in an additional 37 biopsies using both FD and white light imaging. The results are summarized in Figure 3. In 26 of the discrepant biopsies, FD was positive and white light imaging was negative, with 19 true positives and 7 false positives. In the remaining 11 discrepant biopsies in which fluorescence detection was negative and white light imaging positive, there were 8 false negatives for FD, while white light imaging had 8 true positives and 3 false positives. Postoperative complications occurred in 3 patients: 1 died; the second had a wound infection that was treated with oral antibiotics and rinsing of the thoracic cavity; and the third, a pulmonary infection that was also treated with antibiotics.

#### Discussion

Prior studies using animal models have indicated that using 5-ALA in FD may be more sensitive than white light examination and thereby may improve the diagnostic yield.[2–4]In this study, based on fluorescence positive images, 4 of 15 mesothelioma patients were upstaged due to improved detection of lesions. Nonetheless, this study showed that there was no overall improvement observed with the use of FD over white light imaging in establishing a diagnosis. Also, there were 8 biopsies that were positive for mesothelioma under white light imaging which evaded detection by FD. A paucity of data exists on FD using 5-ALA in humans; but of the available photosensitizers, 5-ALA has the advantage of being a naturally occurring nutrient that is metabolized by body cells, with very limited side effects and the ability to be administered both systemically and topically. Another method that can be used for fluorescence detection in the thoracic cavity is the use of porphyrin compounds, but success has been limited in its use[5–7] because significant side effects exist. Autofluorescence and methylated forms of aminolaevulinic acid have also been tried, but they too have inherent drawbacks. The authors surmise that in the future, FD may prove useful in patients presenting with limited pleural disease. Larger trials with a more diverse patient population are needed to confirm this.

#### Conclusion

In the study, though the staging of mesothelioma was facilitated with the use of FD, its overall benefit was limited. It is our conclusion that the combined use of both modalities could provide a better diagnostic yield in the evaluation of undiagnosed pleural disease.

### Positron emission tomography predicts survival in malignant pleural mesothelioma[8]

#### Background

**Figure 4:**
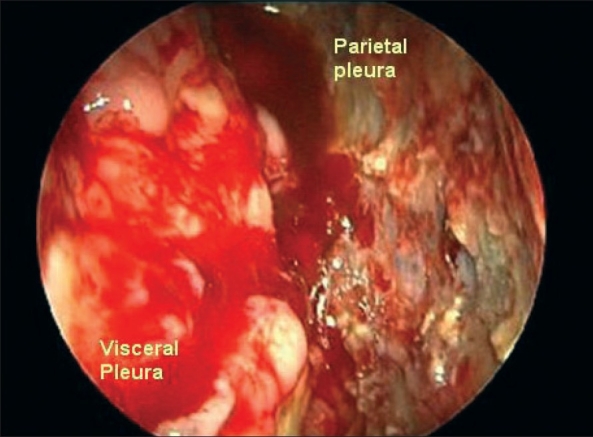
Medical thoracoscopy showing advanced-stage malignant mesothelioma with diffuse tumor involvement of both parietal and visceral pleural surfaces

Malignant pleural mesothelioma (MPM) is a rare disease for which there is no universally accepted treatment. Currently, histology and tumor stage are the accepted predictors of survival for directing treatment and stratifying patients. Video-assisted thoracoscopy remains the gold standard for diagnosis [Figure 4]. Recent studies suggest that standard uptake value (SUV) on fluorodeoxyglucose positron emission tomography scan (FDG-PET) can assist in defining the tumor biology of MPM, such as predicting mediastinal lymph node status. The objectives of this study were to determine whether PET SUV predicted survival and to identify an SUV cut off value that stratifies patients into high- and low-risk groups.

#### Methods

All patients with pathologically proven mesothelioma from April 1998 to January 2005 underwent FDG-PET scanning. Patients fasted and received a minimum of 10 mCi of FDG. An SUV of 10 was chosen to categorize patients into either high- or low-risk groups based on the maximal chi-square method,[9] and survival probabilities for both SUV groups were estimated by the Kaplan-Meier method. A Cox proportional hazards model assessed the combined influence of SUV, histology and stage on survival.

#### Results

A total of 137 consecutive patients (96 men and 31 women) with biopsy-proven, previously untreated MPM underwent FDG-PET scanning as part of their initial staging evaluation. Of these, 106 patients (77%) had tumors of epithelioid histology; 27 (20%), mixed subtype; and 4 (3%) had sarcomatoid subtype. Patients' disease staging was as follows: stage I - 3, stage II - 21, stage III - 47 and stage IV - 56. One hundred thirteen patients underwent surgical procedures, while 19 patients did not undergo any surgical procedure. The median follow-up of surviving patients was 24 months. A near-linear relationship was found, in which increasing SUV correlated with poor survival outcome. The median survival in stage I and II tumors, as well as in those with an SUV of less than 10, was significantly greater than that of stage III and IV tumor patients and those with SUV of 10 or more respectively (*P* = 0.02). Patients with tumors with an SUV of greater than 10, with non-epitheloid tumor histology and with stage III and IV tumors had a higher risk of death than patients with an SUV of less than 10, with epitheloid tumor histology and with stage I and II tumors respectively (*P* < 0.01, *P* < 0.01 and *P* = 0.05 respectively).

#### Discussion

Prior studies have shown that computed tomography (CT) and magnetic resonance imaging (MRI) frequently fail to stage MPM accurately at the time of diagnosis.[10] Several small studies have investigated the utility of FDG-PET scan in mesothelioma but have been largely inconclusive. In this study, there was no significant difference in the median SUV between epitheloid and non-epitheloid tumors, while there were too few patients with sarcomatoid MPM to draw definitive conclusions regarding SUV levels. This study indicates that ‘increasing SUV levels’ appear to be a predictor of poor prognosis. However, patients with an SUV greater than 10 have a 1.9 times greater risk of death than those with an SUV less than 10.

#### Conclusion

This study demonstrated the ability of FDG-PET to stratify patients' survival when a maximal SUV of 10 was chosen to separate patients into good and poor prognostic groups. However, a prior study analyzing 63 patients showed a survival benefit for those with tumors with an SUV of less than 4.[11] From both studies, the number of patients analyzed seem to affect the SUV cutoff; hence a future analysis using a greater number of patients may result in another value to categorize patients. This raises the question whether a SUV cut off value is useful. This study has shown a near-linear relationship between increasing SUV and poor prognosis; therefore, the combination of SUV, histology and stage could provide a convenient and clinical way of identifying high- and low-risk MPM patient groups, thereby directing their treatment or stratifying them for clinical trials.

### Emergency ultrasound evaluation of symptomatic nontraumatic pleural effusions[12]

#### Background

The value of ultrasound (US) for diagnosis of pleural effusion is well documented.[13,14] This study tried to prove that thoracic ultrasound (ThorUS) performed by emergency physicians in symptomatic ED patients would be a rapid and effective management tool for the evaluation of nontraumatic pleural effusion.

#### Methods

This study was a prospective observational investigation of symptomatic adults with suspicion of pleural effusion on chest X-ray. Emergency Department physicians used US for the evaluation of the torso. All EM -attending physicians had met at the American College of Emergency Physicians 2001 Emergency Ultrasound Guidelines for initial emergency US education, and all participating EM-attending physicians were credentialed in emergency US.

Enrolling physicians completed a questionnaire related to patient care both before and after ThorUS.

The examination consisted of identification of several key elements for this study, including (1) the diaphragm (2) the presence or absence of anechoic to hypoechoic collections above the diaphragm and (3) level of highest intercostal involvement.

Treating physicians were surveyed on perceived versus actual procedural time required, pre-and post-test likelihood of pleural effusions using a 10-point scale, diagnostic benefit and changes in management.

#### Results

**Figure 5:**
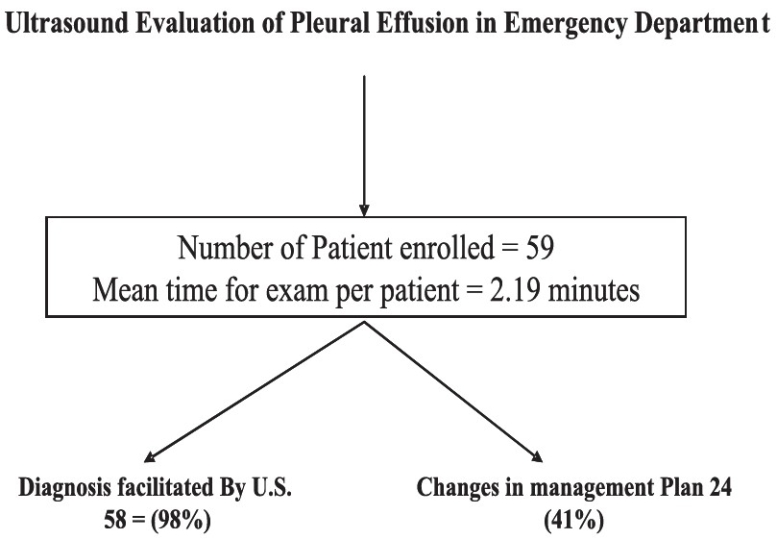
Utilization of ultrasound by trained emergency department physicians facilitated the diagnosis and changed the management in a significant number of patients

Fifty-nine patients (54% males) with an average age of 61 + 17.2 years (SD) were enrolled. Investigating physician's perceived time to perform ThorUS was 3.13 min (95% confidence interval [CI], 3.09-4.03); whereas the actual average time to perform ThorUS was 2.19 min (95% CI, 1.63-2.64). After ThorUS, 48 (81%) cases had an increase and 11 (19%) had a reduction in physician-determined likelihood of pleural effusion. The average absolute difference in physician-determined pre- and post-test likelihood of pleural effusion was 34% (95% CI, 34.0-34.4). The average change in physician-determined likelihood for those confirmed to have pleural effusion by ThorUS was increased by 35% (95% CI, 22.1-42.4), whereas those having pleural effusion excluded by ThorUS decreased by 30% (95% CI, 16.7-42.4). Emergency physicians felt ThorUS assisted diagnosis in 58/59 (98%) patients. US changed physician management in 41% of patients, with the greatest number of changes occurring in the decision to perform thoracentesis (15%). Fifteen patients (25%) underwent thoracentesis with an average drainage of 1,413 + 913 ml, with a range of 300–3,800 ml. Figure 5 summarizes the main findings in an algorithmic form.

#### Discussion

This study focused on the feasibility, decision-making and management of patients. The time to perform ThorUS was generally short. The changes in management were moderate, with the most significant change being performance of thoracentesis. The study indirectly suggested that plain chest radiograph (CXR) may not be specific for most emergency physicians to make decision regarding thoracentesis.

The use of US, however, does not negate the role of plain CXR in the patient's evaluation but rather supplements the clinical and radiographic findings quickly. Addition of a control arm in the study with decubitus CXRs would be useful, since the method remains the most common practice in community emergency room settings. One of the limitations of the study, addressed by the authors, was that all physicians performing the examinations at the center had adequate US experience. This may not represent the experience of other centers. Also, the total time for ultrasonographic evaluation of pleural effusion (whether perceived or actual) may only represent a portion of the time of actual sonographic evaluation, as previously mentioned. The observational study design did not provide a comparative alternative arm of study or a gold standard criterion, thus limiting the ability to generalize regarding diagnostic accuracy. Also, some conclusions made in the study were predicated on the basis of data that was subjective (emergency physicians feeling that ThorUS assisted diagnosis in 58/59 (98%) patients).

#### Conclusion

ThorUS performed by emergency physicians is a rapid and effective management tool for the evaluation of nontraumatic pleural effusion in symptomatic ED patients if used by physicians who have already learnt the technique of the common emergency US application.

### Asbestos exposure, pleural mesothelioma and serum osteopontin level[15]

Retrospective studies of small numbers of patients with pleural mesothelioma have attempted to identify biomarkers that predate symptoms in a high-risk population.

At present, there are no economically feasible, validated methods to screen persons at risk. Osteopontin, a glycoprotein that is over-expressed in lung, breast, colorectal, gastric and ovarian cancer and in melanoma, [16–23]was the most promising biomarker. It mediates cell-matrix interactions and cell signaling and is regulated by proteins in cell-signaling pathways that are associated with asbestos-induced carcinogenesis.

#### Methods

**Figure 6:**
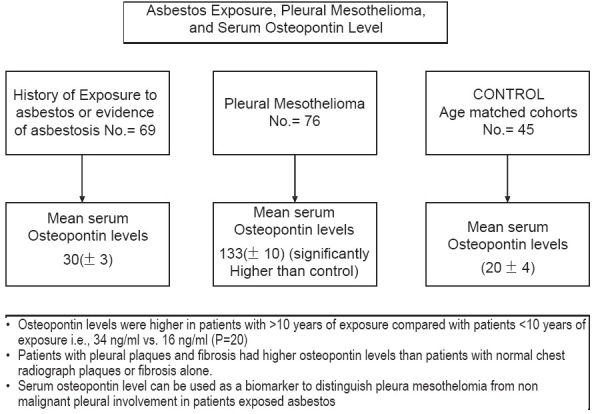
Utility of serum osteopontin levels in distinguishing pleural plaques, asbestosis and malignant mesothelioma

Three groups of subjects were studied [Figure 6]. All subjects provided serum samples.

Sixty-nine subjects were enrolled in the first group with a history of exposure to asbestos, evidence of asbestosis or both. A plain chest radiograph was obtained from each subject and interpreted by a single trained radiologist who was unaware of the patient's exposure status.

There were 45 patients in the second group; of them, 25 current smokers and 20 former smokers underwent screening bronchoscopy as an entry criterion for a chemoprevention trial. All these subjects had no known exposure to asbestos by history, and all their chest radiographs were normal.

There were 76 patients in the third arm; serum was obtained from all these patients. These patients were scheduled to undergo cytoreductive surgery for pleural mesothelioma. All patients underwent complete surgical staging. All were followed with computed tomography of the chest every 3-4 months until death or to a later predetermined follow-up date.

#### Results

The mean serum level of osteopontin in the entire group of subjects who were exposed to asbestos did not differ significantly from that in subjects without exposure to asbestos (*P* = 0.06). The levels in controls with no exposure to asbestos and normal radiographs did not differ significantly according to age from those in the group exposed to asbestos. In the group with exposure to asbestos, there were no significant differences in osteopontin levels according to the presence or absence of pleural plaques (*P* = 0.88). The subgroup with lung fibrosis had a significantly higher mean level of osteopontin than the subgroup without fibrosis (*P* = 0.004), and the mean levels were significantly higher with 10 or more years of exposure than with fewer than 10 years of exposure (*P* = 0.02). The highest levels of serum osteopontin were found in subjects who had both plaques and fibrosis. Among the subjects with exposure to asbestos, osteopontin levels were significantly lower in subjects with a normal chest radiograph, subjects with plaques and subjects with fibrosis than in those who had plaques and fibrosis (*P* = 0.004).

The duration of exposure to asbestos and the radiographic findings were independently associated with osteopontin levels (*P* = 0.001 and *P* < 0.001 respectively).

The mean serum osteopontin level in the group with pleural mesothelioma differed significantly from that in the group exposed to asbestos (*P*< 0.001). Furthermore, serum osteopontin levels in the subjects with exposure to asbestos with plaques and fibrosis differed significantly from those in the patients with pleural mesothelioma (*P*<0.001). Mean osteopontin levels were similar in men and women with mesothelioma (*P* = 0.66) and did not vary according to the histologic characteristics of the tumor (*P* = 0.49).

#### Discussion

The authors conclude from this study that serum osteopontin levels can be used to distinguish persons with exposure to asbestos with benign disease from those who have pleural mesothelioma. They also concluded that the most important result of the study was the apparent ability of an enzyme-linked immunosorbent assay (ELISA) for osteopontin to identify early pleural mesothelioma (stage I).

Cullen in his editorial review of the study believes that the practical value of the use of serum osteopontin levels as a diagnostic test must await the assessment of similar measurements in other disorders common after extensive exposure to asbestos, such as diffuse pleural thickening with or without benign effusion, advanced carcinoma of the lung with pleural involvement and rounded atelectasis, from which mesothelioma must commonly be differentiated.[24]

#### Conclusion

Measuring serum osteopontin could be an important step in early diagnosis and management of mesothelioma in the future.

## II. TREATMENT

### Fibrinolytics

**UK controlled trial of intrapleural streptokinase for pleural infection multicenter, intrapleural sepsis trial (MIST 1)[25]**

#### Background

Approximately 65,500 patients in the USA and UK develop pleural infections, of which 15% die and an additional 15–40% require surgical drainage of the infected pleural space.[26,27] Small trials and case series suggest possible benefits from the intrapleural administration of fibrinolytic drugs such as streptokinase.[28] Prior studies performed had a low statistical power. In an evidence-based guideline, poor surgical candidates with multiloculated effusion were candidates to receive fibrinolytics.[29,30]

The aim of the current trial was to accurately assess the benefits of intrapleural streptokinase in the treatment of pleural infection.

#### Methods

A double-blind, randomized multicenter trial was performed in 52 centers consisting of 27 teaching and 25 community hospitals. The inclusion criteria for the trial was the presence of pleural fluid, characterized by being visibly purulent during drainage, positive for bacterial infection on culture, positive gram stain or with pleural pH <7.2 with clinical evidence of infection (i.e., fever, elevated white cell count or elevated serum level of C-reactive protein). All patients underwent chest tube drainage and received intravenous antibiotics. The patients were randomly assigned to two treatment groups to receive either 250,000 IU of streptokinase or placebo via the chest tube every 12 h for 6 doses. The primary end points were the number of patient deaths or patients requiring surgical drainage of infected pleural fluid within 3 months post-randomization. The secondary end points were death or surgical drainage 12 months post-randomization, the duration of hospital stay, the severity of residual abnormality on the chest radiograph (including dynamic lung volumes at 3 months post-randomization), bleeding occurring in the post-surgical period, successful drainage of empyema and changes in anti-streptokinase antibody levels from baseline to 3 months. Patients who did not receive the assigned study drug because of death, surgery or withdrawal of consent were excluded from the primary analysis.

#### Results

**Figure 7:**
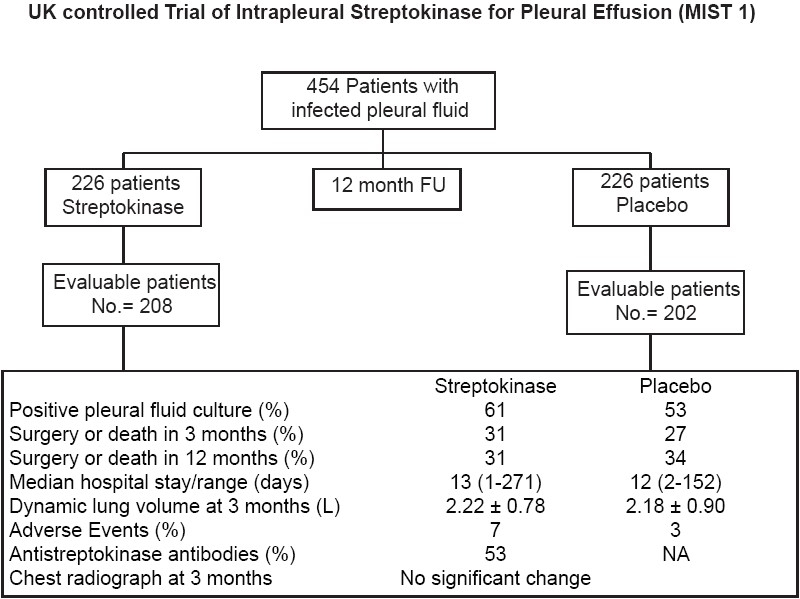
The usefulness of streptokinase instillation in empyemas was evaluated over a 12 month period. There were no significant differences in the primary endpoints studied over a 3 and 12 month period

The results are summarized in Figure 7. A total of 454 patients were recruited for the trial. Twenty-four patients either died, required surgery or withdrew their consent before receiving the study drug. The baseline characteristics of the two study groups were similar, with 430 subjects being analyzed. The test group comprised of 208 of the analyzed subjects who received streptokinase while the control group (n = 222) received placebo. In each group, 206 patients completed the trial. The primary outcomes at 3 months post-randomization showed no statistically or clinically significant difference between the two study groups in the proportion of patients who required surgical drainage or those who died. The secondary outcomes showed no statistically significant difference in the proportion of patients who required surgical drainage or those who died at 12 months post-randomization. Similarly, there was no difference between the two study groups at 3 or 12 months when death and the need for surgical drainage were analyzed separately. There was no difference in the duration of hospital stay and in the degree of pleural thickening at 3 months. However, there was a small but statistically significant difference in favor of placebo in the height of fluid column/empyema on the ipsilateral side at 3 months. There was no difference in the degree of reduction of the size of pleural opacity at 3 months, in the spirometric lung volumes in the two groups at 3 months or the need for blood transfusions in those patients who bled after surgical drainage.

#### Discussion

The conclusions of this study were weakened by the enrollment of a heterogeneous patient population at different stages of empyema formation, the lack of staging of the empyema, the advanced age of many patients and large proportion of patients with coexisting conditions, which could have influenced some of the study end points. The important interventions such as the administration of antibiotics and techniques for the insertion of chest tubes were left to the judgment of individual clinicians and were not pre-specified in standardized protocols. The referral for surgical drainage was also based on individual clinical judgment.

It is suggested that streptokinase did not improve long-term outcomes in this trial because it did not improve the fluid characteristics of viscosity or lower resistance to tube drainage. Streptokinase is believed to simply breech the barriers between loculations but failed to drain adequately as the fluid was unable to pass down the chest tube. It is believed that reducing the fluid viscosity with the use of DNAase in combination with fibrinolytics to disrupt loculations may improve outcomes in pleural infection, as streptokinase alone does not produce sufficient clearance of pleural fluid. In an *in vitro* study performed by Zhu *et al.,*[31] the combination of alteplase and rhDNase is more effective in the treatment of rabbit empyema than either agent alone.

#### Conclusion

In this study, there was no evidence of reduction in the need for surgical drainage, mortality or the length of hospital stay after the administration of streptokinase. The use of intrapleural streptokinase should therefore be generally avoided in pleural infection. Appropriate trials are needed to explore the possibility of improved outcomes in pleural infection treatment using a combination of fibrinolytics and Dnase.

### Intrapleural fibrinolytic agents for empyema and complicated parapneumonic effusions: A meta-analysis[32]

#### Background

Previous small trials have suggested the benefits of fibrinolytic agents. However, the recent MIST 1, the largest trial to date, revealed no improvements in terms of death, rate of surgery, radiographic outcome and duration of hospital stay. This meta-analysis tried to clarify the current role of intrapleural administration of fibrinolytic agents in the treatment of empyema and complicated parapneumonic effusions.

#### Materials and Methods

Literature searches were performed to identify all relevant published and unpublished randomized controlled trials (RCTs) from January 1980 to March 2005 comparing intrapleural fibrinolytic agents with placebo for the treatment of pleural infection (empyema and complicated parapneumonic effusions). The MIST 1 trial was also included in the analysis. A search was extended to any language of publication and limited to studies involving only humans.

Two investigators independently evaluated studies for inclusion, and the disagreements were referred to a third investigator. Criteria for inclusion were as follows: (1) randomized trials, (2) allocation concealment, (3) objectively diagnosed empyema or complicated parapneumonic effusions, (4) comparison of fibrinolytic agents with placebo and (5) objective methods to assess clinical outcomes. Trials involving patients who had prior surgical intervention, posttraumatic infection and those conducted in children were excluded from this analysis. The primary outcome chosen was the reduction in both mortality and the need for thoracic surgery. The secondary outcomes were the individual components of the primary outcome, the duration of hospital stay and improvement in chest radiography.

#### Results

Three hundred fifty-six potentially eligible studies were identified. Only five RCTs were included for meta-analysis.[33–35] The ATS guidelines were used to classify stages of empyema.[35] The inclusion criteria for entry were slightly different amongst the different trials included in the final analysis. Also to be noted is that the meta-analysis also included patients with tuberculous pleuritis (n = 3; 7%).

Four trials suggested a reduction in mortality and a reduction in the need for surgical intervention by intrapleural fibrinolysis compared with placebo. It should be noted that these four trials were much smaller (n = 148) as compared to the MIST 1 trial (n = 430). However, the pooled estimate of relative risk (RR) from all of the five trials was not statistically significant (treatment group, 27.6% *vs.* control group, 32.8%; RR, 0.55; 95% CI, 0.28-1.07).

Likewise, when the need for surgery was separately analyzed, a significant heterogeneity was noted. Therefore, using a random-effects model, a nonsignificant treatment effect was obtained (treatment group, 15.8%; control group, 22.3%; RR, 0.71; 95% CI, 0.28–1.02). The pooled estimation for mortality was analyzed from only two trials since the remaining three trials showed no mortality identified in both groups. No patients experienced side effects in four trials, except in MIST 1,[36] in which the treatment group had serious adverse events in 7% and the control group in 3% (unpaired *t* test, *P* = 0.08 by their analysis).

#### Discussion

This meta-analysis was published a few months after the MIST 1 study. It provided no evidence of benefit of intrapleural fibrinolytic therapy for reduction of mortality and the need for surgery in adult patients with empyema and complicated parapneumonic effusions. Like most meta-analyses, there were differences in the respective study designs.

The small-sample-size RCTs suggested positive benefits, while the recently published large trial, MIST 1, showed negative results. The reasons for this dichotomy may be multifactorial. In MIST 1, streptokinase was mailed to study centers after randomization. This may have delayed fibrinolytic treatment that might have been potentially effective if used in a timely manner. Furthermore, MIST 1 used relatively smaller chest tubes (median, 12F) without ultrasonographic guidance. Also, decisions for the need of surgical intervention were based on clinical judgment without objective protocols, adding a potential bias across all of the trials. The duration for assessing outcomes was provided only in MIST 1, and possibly this duration (3 months) might have been relatively longer than in other trials to assess mortality. Finally, in MIST 1, mortality in both groups was higher than that of the other trials.

These trials should not be construed as a moratorium for intra-pleural thrombolytic therapy. Intrapleural fibrinolytic therapy may still have some role, especially if used in conjunction with newer drugs, such as deoxyribonuclease, which reduces the viscosity of pleural purulence and promotes its drainage. In addition, fibrinolytic agents may be attempted in patients with persistent sepsis who are poor candidates for surgical drainage due to serious comorbidities.

#### Conclusion

The limitations of this study, as pointed out by the authors, stem from the limited number of trials and especially the limited number of patients if the MIST 1 trial is excluded. Therefore, the conclusions of this study do not differ from those reached by the MIST study discussed earlier.

## Pleurodesis agents

### Prospective randomized trial of silver nitrate *vs.* talc slurry in pleurodesis for symptomatic malignant pleural effusions[37]

#### Background

Almost 50% of patients with malignant pleural disease have an associated malignant pleural effusion. Breast and lung cancers are responsible for approximately 75% of these effusions.[38] Dyspnea and cough are the most common symptoms secondary to malignant pleural effusion. If symptoms are relieved with therapeutic thoracentesis and full re-expansion of the lung is observed, then it is reasonable to consider pleurodesis. Currently, this is most commonly done with the injection of a sclerosing agent through a chest tube.

Spengler likely performed the first pleurodesis in the beginning of the 20^th^ century.[39] He injected silver nitrate (SN) into the pleural space to control recurrent pneumothorax. A recent study using 0.5% silver nitrate in rabbits resulted in good pleurodesis.[40] Beginning in 1935, talc was used as a sclerosant. It became popular because it was cheap, easy to handle and available worldwide. It was well tolerated, with few side effects and was effective in >90% of the patients.[41] In the 1980s, several cases of acute respiratory distress syndrome (ARDS) were attributed to talc.[42] After silver nitrate and talc, multiple other agents have been used in attempts to create a pleurodesis. However, the ideal sclerosing agent has yet to be identified. The ideal properties include low cost, ready availability, ease of use, ease of introduction into the pleural space and being devoid of significant side effects.

#### Methods

Sixty patients with unilateral malignant pleural effusion were enrolled into this study. Inclusion in the study required documentation of a malignant pleural effusion (i.e., positive result of testing of pleural biopsy specimen or cytology), a Karnofsky index score of >60 and a life expectancy >1 month. Patients with loculated effusions or those who had trapped lungs after drainage were excluded from the study. Eleven of the patients did not return for their follow-up visit and were excluded from the analysis.

In all patients, a chest tube (26F or 28F) was inserted. The drainage of the fluid was slowed when the patient experienced coughing or chest tightness to avoid re-expansion edema. The patients were randomized to receive either talc or SN through the chest tube. The pleural sclerosant was then injected. The pleural injectate consisted of 5 g sterilized talc diluted to a total volume of 50 ml with saline solution or 20 ml 0.5% SN. The talc preparation was asbestos-free with a mean fiber length of 25.4 μm (range, 6.4-50.5 μm). After the sclerosant was injected, the chest tube was clamped for 1 h and the patient was placed in the prone, supine and right and left decubitus positions for a period of 10–15 min in each position. The chest tube was then unclamped and placed on - 20 cm H_2_O suction. Serial radiographs were used to document appropriate lung re-expansion. The patients were followed up every day, and the chest tube was removed when the amount of fluid collected in the prior 24 h was <100 ml. They were clinically evaluated before and after treatment regarding pain using a linear scale of 0 to 10. Chest radiographs were obtained immediately following tube removal and at their monthly follow-up visits. Patients were requested to return at monthly intervals for 4 months. At these visits, patients were considered to have a successful pleurodesis if there was no recurrence of the pleural effusion. Patients who did not have a pleurodesis at one of their visits were excluded from subsequent visits.

#### Results

Sixty patients received an intrapleural injection with talc or SN. Eleven patients were excluded from the study because they were unavailable for follow-up. The remaining 49 patients were considered to be appropriate for analysis because they were seen for a follow-up visit and had undergone a chest radiograph at least 30 days post-’pleural injection’. The groups were similar in age, sex, Karnofsky index score and pathology (*P* = 0.68).

Following the treatment, subjective pain was low and similar with both sclerosants including on day 5. The total fluid drainage after the induction of pleurodesis was not statistically different between SN (901 ± 125 ml) and talc (766 ± 74 ml). The mean number of days spent in the hospital was nearly the same in the group that received intrapleural SN (3.7 ± 0.15 days) and in the group that received talc (3.6 ± 0.13 days). None of the patients who returned for follow-up had significant long-term adverse affects from the pleurodesing agents. None of the patients in the talc group developed ARDS.

**Figure 8:**
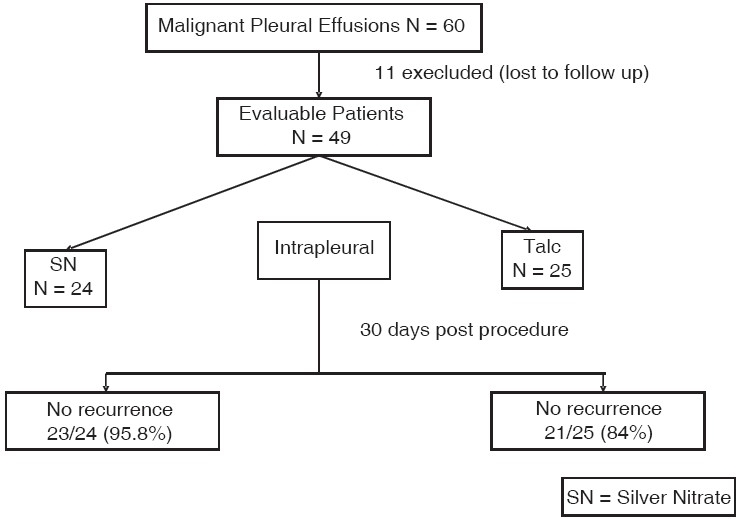
Comparison was made in the response rates of Silver Nitrate and talc pleurodesis in malignant pleural effusions. The results were not significantly different among the two groups

Thirty days after undergoing the procedure, 23 (96%) of 24 patients who had received intrapleural SN and 21 (84%) of 25 patients who had received talc did not have recurrence. There was no statistically significant difference between the groups (*P* = 0.349). Similar results were observed after 60 days, 90 days and 120 days. These findings are summarized in Figure 8.

#### Discussion

The study was not blinded. It was also underpowered to detect differences in SN versus talc with respect to primary and secondary outcomes. There were more breast cancer patients in SN group (22 *vs.* 14) as compared to the talc group. A total of 11, or nearly 20%, patients were lost to follow-up. The authors did not report reasons but acknowledge that most patients were in the SN group. Conclusions regarding the long-term safety could not be made.

#### Conclusion

Although the authors state that silver nitrate appears to be at least as effective as talc, at this stage we can only recommend that SN as an alternative to talc is worth considering. However, more studies are needed regarding the long-term effects of silver nitrate. This question may be more relevant in those who have a longer life expectancy, such as patients with a spontaneous pneumothorax versus those with a malignant pleural effusion.

### Randomized trials describing lung inflammation after pleurodesis with talc of varying particle size[43]

#### Background

Many of the pleural effusions caused by cancers are symptomatic, requiring the use of pleurodesis for the control of symptoms. Sterile talc is considered the most effective agent for this procedure. The success rate for pleurodesis is approximately 93% compared to 72% for doxycycline and 54% for bleomycin.[41] Safety issues with talc pleurodesis have surfaced since there have been cases of acute respiratory distress syndrome (ARDS), some of which resulted in death.[44] The toxicity following intrapleural talc administration is thought to correlate with the small (<15 μm) size of the talc particles, but there have been no randomized trials assessing the potential toxicity of varying talc preparations. This article reports two randomized trials addressing this question.

#### Methods

Two prospective parallel randomized trials were performed in a single center where patients with proven or suspected malignant pleural effusions were referred. The entry criteria for the two studies were the same and included patients with symptomatic pleural effusions proven to be due to pleural malignancy. The exclusion criteria included expected survival of less than 6 weeks, bleeding diathesis sufficient to contraindicate chest tube insertion, extensive trapped lung after fluid drainage, previous pleurodesis on the side of the effusion, inability to give informed consent or age less than 18 years. Patients were followed for 3 months or until death.

##### Trial 1

Subjects were randomized to receive either mixed talc (50% particles <15 μm) or tetracycline for pleurodesis. The primary outcome was the change in technetium-labeled diethylene-triamine-penta-acetic acid (DTPA) clearance from the contralateral lung from baseline to 48 h after the procedure. The secondary outcomes included arterial oxygen saturation sitting in a recumbent position and breathing air, plasma C-reactive protein (CRP) levels, pleural fluid interleukin (IL)-8 concentrations and the presence of visible ground glass shadowing on high-resolution thoracic computed tomography (HRCT) from baseline to 48 h after the procedure.

##### Trial 2

Subjects were randomized to receive either mixed or graded talc (50% particles >25 μm) for the pleurodesis. The primary outcome was the change in alveolar to arterial (A-a) oxygen gradient from baseline to 48 h after the procedure. The secondary outcomes included arterial partial pressure of oxygen (P_a_ O_2_), fever >37.5°C at 48 h, the plasma CRP, pleural IL-8 concentrations and the clinical efficacy of pleurodesis assessed at 3 months after pleurodesis.

#### Results

##### Trial 1

Thirty-one subjects were recruited and they consented to the study; of them, 11 individuals were excluded. Thus, 20 patients were randomized to either talc or tetracycline and they completed the study.

With regard to the primary end point, the fall in the isotope clearance half time was greater in the mixed-talc group as compared to the tetracycline group {-9.26 [SD 14.3] *vs.* 4.10 [SD 13.8] min (*P* = 0.05)}. The fall in clearance was indicative of clinically significant lung inflammation and impairment of gas exchange. Evaluation of the secondary end points revealed that when comparing the mixed talc and tetracycline groups, the fall in arterial oxygen saturation and CRP rise were greater after mixed-talc pleurodesis, indicating a greater systemic inflammatory response to mixed talc compared to tetracycline. Finally, there was no difference in IL-8 levels or HRCT findings in either trial group.

##### Trial 2

A total of 56 subjects were recruited and they consented to the study, with 10 of them being excluded. Forty-six patients were randomized to either graded or mixed talc, and all of them completed the study.

Review of the primary end point revealed that mixed-talc pleurodesis resulted in a greater increase in the (A-a) oxygen gradient than graded-talc pleurodesis, indicating a greater deterioration in blood oxygenation. Similarly, review of the secondary end points revealed that with mixed-talc pleurodesis, fall in PaO_2_ was greater, more subjects had a fever >37.5°C (41% versus 4%) and CRP rise was greater when compared to graded-talc pleurodesis. Also, there was no difference in IL-8 levels or in the success of pleurodesis (79% success in mixed versus 85% in graded).

#### Discussion

This study suggests that pleurodesis-induced hypoxemia results from generalized lung inflammation, as well as from an increase in systemic inflammation. The authors concluded that mixed-talc pleurodesis produces more lung inflammation and systemic inflammation and leads to worsening hypoxemia as compared with graded-talc (or tetracycline) pleurodesis. They postulated that many patients experiencing clinically significant hypoxemia may have reduction in their symptoms with the use of graded talc. It should be pointed out that although there was a trend towards a fall in the isotope clearance half time in the mixed-talc group as compared with the tetracycline group, the ‘*P*’ value achieved was 0.05. Multiple comparisons for significance were studied in both trials. Correction for multiple comparisons in Trial 1 left only changes in arterial oxygen saturation as significant between the test and the control groups. Similarly, correction for multiple comparisons in Trial 2 left only fever at 48 h as significant.

Experience gained from one trial was utilized to modify the second trial. The first trial used DTPA clearance as the primary end point. At the end of this trial, it was felt to be a complex investigation and hence was dropped from the second trial.

A multicenter, open-label prospective study was recently published.[45] The objective of the study was to establish whether use of large-particle-size talc is safe in patients with malignant pleural effusion. The primary end point was the occurrence of ARDS after pleurodesis. Secondary end points included other adverse outcomes and death within 30 days. A total of 558 patients were recruited. Seven patients developed a new infiltrate. One patient developed respiratory failure not caused by ARDS. A significant increase in mean temperature was seen on days 1 to 4 after the procedure as compared with the baseline. No significant difference in oxygen saturation was noted after the procedure. However, there was a significant rise in the supplemental oxygen requirements as recorded for the first two days after the procedure compared with baseline. Eleven patients (2%) died within 30 days (range 2-29 days, mean 11.8 days) after the procedure.

This study supports the hypothesis that ARDS and severe hypoxemia can be avoided by use of large-particle talc. The study had a large number of patients but was not controlled with small-particle talc group to assess both safety and efficacy. This was because it was felt that it might be unethical, especially with growing evidence suggesting that large-particle talc may be safer.

#### Conclusion

This study demonstrated significant lung inflammation associated with a greater degree of hypoxemia following mixed-talc pleurodesis as compared to graded-talc pleurodesis. Given the potential benefits of the latter, routine use of graded talc is recommended to reduce the morbidity of this procedure.

### Simple aspiration versus chest tube insertion in the management of primary spontaneous pneumothorax: A systemic review[46]

#### Background

Primary spontaneous pneumothorax (PSP) was first described in 1932.[47] Its global incidence is estimated at 18-28 per 100,000 for men and 1.2-6 per 100,000 for women.[48,49]

The American College of Chest Physicians (ACCP) guidelines recommend chest tube (CT) or pleural catheter as the preferred intervention over simple aspiration (SA).[50] They advocate SA only for stable patients with small PSP that progress with observation.

The British Thoracic Society (BTS) guidelines recommend SA for all PSP requiring intervention, regardless of the size of the pneumothorax.[49]

Seeing that available data is limited, this systemic review was undertaken to determine an objective estimate of which is the better treatment option based on evidence from RCTs.

#### Methods

The authors conducted a meta-analysis of RCTs using Medline 1966 to June 2004, EMBASE 1974 to June 2003 and Cochrane Central Database. The search was limited to RCTs that compared SA and CT in any language.

#### Results

Three RCTs from Europe and North America fulfilled selection criteria.[51–53]

When the data from the three trials were pooled, patients treated with SA had a shorter duration of hospitalization (-1.30 days). There was no statistically significant difference in success (near-complete re-expansion of the lungs and cessation of any air leak) at 1 week between SA and CT. There was no significant difference between SA and CT in the recurrence rate reported after 3 months in one trial and at 1 year in the other two RCTs.

#### Discussion

None of these studies described concealment of allocation or blinding. Consequently, selection and performance bias were not eliminated. While blinding treating physicians for chest tube insertion or aspiration of pneumothorax is practically difficult, it might still have been possible to blind study evaluators who were deciding the success and failure of the modalities being compared in these trials.

The meta-analysis suffered from the usual problems with such reviews: lack of description of the critical design elements; patients not limited to PSP; delay in performance of SA by 72 h in one study; or recruitment of patients only with first occurrence of PSP. The authors concluded that RCT evidence in this field is limited, total sample size is too small to make any firm conclusion and SA is advantageous because of shorter hospitalization.

#### Conclusion

At this stage, we would not recommend modifying any practice patterns based upon this meta-analysis.

### Single-center experience with 250 tunneled pleural catheter insertions for malignant pleural effusion[54]

#### Background

**Figure 9:**
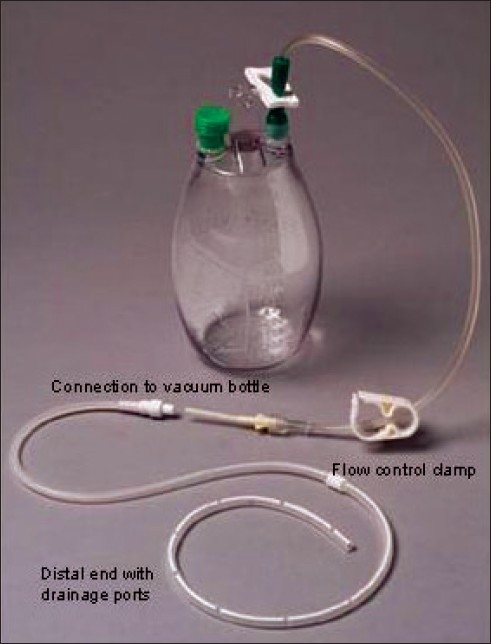
The silicone catheter has multiple ports at the distal end to allow drainage of pleural fluid. The proximal end has a one-way valve and is connected to a vacuum bottle with a custom connection. Patient can control the flow of drainage with the clamp. Courtesy: Denver Biomedical

**Figure 10:**
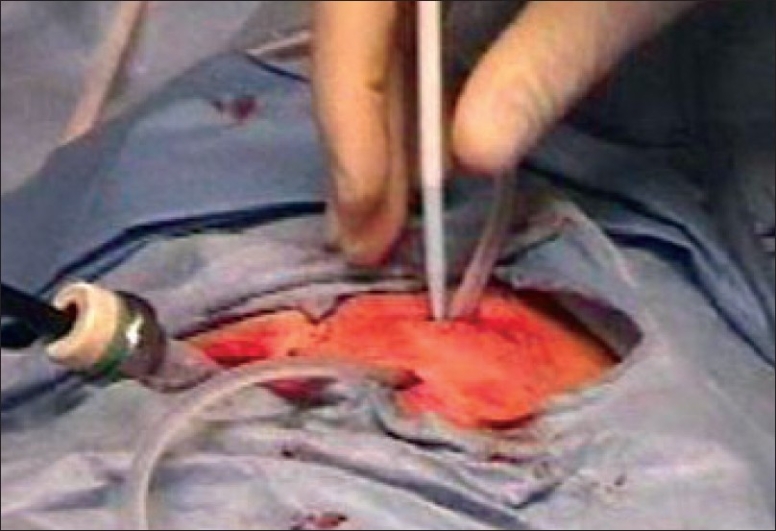
Insertion of tunneled pleural catheter during medical thoracoscopy. A trocar is inserted on the left side, allowing a pleuroscope to directly visualize the pleural surfaces as well as observe the insertion of the tunneled catheter. A introducer is seen being inserted into the pleural space, allowing the distal end of the tunneled catheter to be placed properly. Courtesy: David Feller-Kopman, M.D., Beth Israel Deaconess Medical Center, Boston, MA

The use of a chronic indwelling tunneled pleural catheter (TPC) has gained popularity over the past few years [Figure 9].[55,56] In this study, the authors report their experience with this technique and perform a retrospective analysis of the first 250 TPC insertions for the treatment of malignant pleural effusion (MPE) performed at their center. In many other centers, tunneled pleural catheters can be inserted with medical thoracoscopy [Figure 10] or blindly with Seldinger's technique.

#### Materials and Methods

The insertion of TPCs in this center was performed by pulmonary physicians. All patients were scheduled for an initial follow-up visit and chest radiograph (CXR), followed by the same every 6 to 8 weeks thereafter and on an as-needed basis.

Home care nursing support was organized for all patients to assist TPC care and drainage. TPCs were removed once the volume of fluid drained was <50 ml on three sequential drainages.

Spontaneous pleurodesis (SP) was said to occur when the drainage volume decreased to <50 ml of fluid for three consecutive drainage attempts without progressive symptoms or the re-accumulation of fluid on a CXR. Dyspnea control was determined at the 2-week follow-up evaluation. The patient was deemed to have an adequately re-expanded lung if ≤20% of the treated hemithorax contained fluid.

#### Results

Two hundred fifty TPC procedures for the management of patients with MPE were performed in 223 patients. Symptom control at the 2-week follow-up visit was recorded as ‘complete’ following 97 procedures (38.8%), partial in 125 procedures (50%) and none in 9 procedures (3.6%). In addition, 10 TPC insertions (4.0%) failed, and in 9 other cases (3.6%) symptom control could not be assessed because the patient died within 2 weeks or could not attend the follow-up appointment. The size of the effusion was significantly decreased from baseline to the 2-week follow-up visit (61 to 23%, overall, respectively; *P* < 0.001 [paired *t* test]).

SP occurred following 103 (42.9%) of the 240 successful TPC procedures. Only 2.9% of patients eventually received a sclerosing agent through the TPC. Fifteen additional catheters (6.3%) were removed for various reasons (empyema 5; subcutaneous emphysema 1; symptomatic loculation 3; dislodged 3; ‘trapped lung’ with no improvement in symptoms 1; pain 1; and extrapleural placement 1).

One hundred ten TPCs (45.8%) stayed in place until death. Overall, the TPC remained in place for a median of 56 days until death or TPC removal.

Complications related to the TPC include one episode of recurrent effusion and one case of empyema.

#### Discussion

As compared with chest tube pleurodesis and medical thoracoscopy, TPC is less costly, mitigates the need for hospitalization, can be performed as an outpatient procedure and is safer in the severely debilitated patients who cannot tolerate thoracoscopies.[57–59]

The large majority of TPC insertion procedures lead to symptom improvement in these patients, which is the primary goal of treatment for patients in this condition. It has been shown in a randomized study that TPC and doxycycline pleurodesis lead to equivalent symptom control.[57] There are, however, at least two other studies showing that talc pleurodesis is superior to doxycycline.[60,61]

Based on these studies, we can indirectly conclude that talc pleurodesis is superior to TPC. The procedure is safe, with the most severe complications being empyema (occurring in 3.2% of cases).

Although this study represents the largest group of patients who have been treated with TPC, it is still a retrospective study and has no control group. We agree that TPCs might have significantly lower direct procedure-related costs; but as indicated by the authors, TPCs lead to new expenses such as catheter-draining supplies and home-care support, which are considered postprocedure costs. There is no long-term economic analysis comparing the use of TPCs with other modalities.

#### Conclusion

TPC is an important palliative modality in treating patients with MPE, especially debilitated patients or patients with trapped lung who with drainage. However, it is difficult to agree with the authors to consider TPC as the first line treatment option in patients with MPE. Further studies randomizing patients to treatment with a TPC *vs.* talc slurry or thoracoscopy, including efficacy, complication rate and an economic analysis, would be of benefit.

### A randomized trial of single-dose radiotherapy to prevent procedure tract metastasis by malignant mesothelioma[62]

#### Background

Prior studies have demonstrated that a prophylactic three-fraction course of irradiation completely obviates procedure tract metastasis (i.e., from up to 40% with Abram's needle, thoracic drains/thoracoscopy and fine needle aspiration to 0% after irradiation.[63,64] British Thoracic Society (BTS) guidelines for the management of malignant pleural effusions recommend that patients with proven or suspected mesothelioma should receive prophylactic radiotherapy to the site of biopsy or chest drain insertion. This trial was carried out to test the more convenient single-radiation treatment in the prevention of procedure tract metastasis.

#### Methods

The inclusion criteria for subjects consisted of a histological confirmation of malignant pleural mesothelioma, age greater than 18 years and a clearly identifiable procedure site. The patients were randomized to receive either a single dose of electron beam chest wall irradiation or no prophylactic therapy. The irradiation was given within 15 days of the procedure, and the procedural regions were assessed clinically for masses at 3 and 6 months and subsequently every 6 months until the patient's death. The primary outcome of the trial was the occurrence of procedure tract metastasis. Secondary outcomes consisted of overall survival, tract metastasis rate by procedure type and acute and late radiation toxicities. All analyses were performed on an intention-to-treat basis.

#### Results

**Figure 11:**
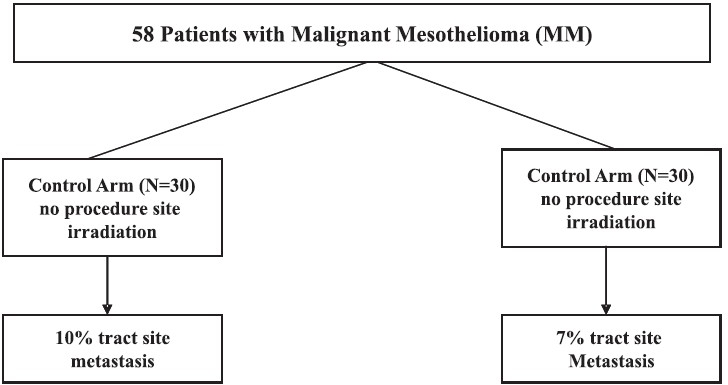
Effect of radiation at procedure site in malignant mesothelioma

A total of 58 procedure sites were registered into the trial, with 28 sites randomized to the prophylactic chest wall irradiation arm and 30 sites to the control arm (no irradiation). There was no statistically significant difference in tract metastasis in the two arms, as demonstrated by a 7% and 10% metastasis rate in the irradiation and control arms respectively [Figure 11]. The secondary outcomes demonstrated no survival difference between arms, no statistically significant difference in tract metastasis by type of procedure performed and mild toxicity in the irradiation group.

#### Discussion

The metastasis in the control arm was low (10%) whereas the median rate in literature, following thoracoscopies, is higher (19%). Many of the procedures performed consisted of fine-needle aspiration, which carries the lowest risk of malignant seeding, and this may have contributed to the low complication rate of 10%. Patients should have been further subdivided into groups according to the type of procedure performed, as the risk of seeding seems to be related to the procedure and technique. The trial should have included an arm consisting of a three-fraction course of irradiation (the current standard of care) to be compared with the single-dose irradiation and no-irradiation group.

#### Conclusion

Even though there is limited information supporting the use of postdiagnostic procedure prophylactic irradiation in mesothelioma, this trial should not currently alter the current practice of utilizing three-fraction irradiation in the prevention of procedure tract metastasis.

#### Summary

This article reviewed some of the important studies in the last few years. The take-home messages from these studies can be concluded as follows:

- Use of fluorescence diagnosis using 5-aminolaevulininc acid, in addition to white light, could provide a better diagnostic yield in the evaluation of undiagnosed pleural disease.
- Fluorodeoxyglucose positron emission tomography (PET scan) can be used with other parameters to stratify patients with malignant pleural mesothelioma for survival.
- Ultrasound could be a useful tool for the evaluation of nontraumatic pleural effusion.
- With further confirmatory studies, osteopontin could be an important step in early diagnosis and management of mesothelioma.
- The use of intrapleural streptokinase generally is not recommended in pleural infection.
- Silver nitrate may be considered an alternative to talc for pleurodesis in patients who do not have long life expectancy.
- Routine use of graded talc is recommended to reduce the morbidity associated with pleurodesis.
- Although simple needle aspiration for treatment of primary spontaneous pneumothorax was found to be associated with shorter hospital stay compared with chest tube, there is paucity of data to support use of one over the other.
- Tunneled pleural catheter is an important palliative modality in treating patients with MPE, especially when pleurodesis in not indicated or contraindicated.
- Prophylactic three-fraction irradiation to the site of biopsy or chest drain insertion is still routinely recommended for patients with proven or suspected mesothelioma to prevent procedure tract metastasis.
